# Supplementary material for: Testing for Benford’s Law in very small samples: Simulation study and a new test proposal
Source: PLoS One. 2022 Jul 22;17(7):e0271969. doi: 10.1371/journal.pone.0271969 (PMC9307211; doi:10.1371/journal.pone.0271969)
Supplement: Attachment — Submitted filename: ReplyReviewer2.docx [file pone.0271969.s002.docx]

***Reviewer #2:*** *A few minor suggestions/corrections: p 10 line 198 and figures 1 and 2, umlaut ü on Hürlimann; p 12 line 221 "not necessarily"; p 16 line 282 "almost 15% of the EU"; references 3 and 4 have no "et al"; references 1,2,14,26,30, 30 are missing capitals on journal titles; reference 32 should read "combining p-values".*

Thanks a lot for spotting these mistakes. They are all fixed in the new version of the manuscript.
